# Effects of postoperative knee bracing on knee function and stability after anterior cruciate ligament reconstruction: A systematic review and meta‐analysis

**DOI:** 10.1002/ksa.70098

**Published:** 2025-10-27

**Authors:** Qitai Lin, Zehao Li, Meiming Li, Xueding Wang, Qian Li, Xingguang Hou, Yongsheng Ma, Wenming Yang, Yugang Xing, Donglin Wang, Fan Yang, Wangping Duan, Xiaochun Wei

**Affiliations:** ^1^ Department of Orthopaedics Second Hospital of Shanxi Medical University Taiyuan China; ^2^ Shanxi Key Laboratory of Bone and Soft Tissue Injury Repair Taiyuan China; ^3^ Academy of Medical Sciences Shanxi Medical University Taiyuan China

**Keywords:** anterior cruciate ligament reconstruction, knee brace, knee function, joint stability, meta‐analysis

## Abstract

**Purpose:**

The use of knee braces following anterior cruciate ligament reconstruction (ACLR) remains contentious. Although frequently prescribed in clinical settings, their effectiveness in enhancing postoperative recovery is uncertain. This study aimed to determine whether postoperative bracing after ACLR confers clinical benefits regarding knee function scores, pain, muscle strength, and joint stability, through a systematic review and meta‐analysis.

**Methods:**

A comprehensive search of PubMed, EMBASE, and the Cochrane Library was conducted through March 2025 to identify randomized controlled trials and case‐control studies evaluating postoperative bracing after ACLR. Meta‐analyses were performed using Review Manager (version 5.3) for the following outcomes: International Knee Documentation Committee (IKDC) objective score, Lysholm score, Tegner activity score, visual analogue scale (VAS) pain score, single‐leg hop test, and side‐to‐side knee laxity. Bias risk evaluation was performed applying the Cochrane Risk of Bias Tool and the Newcastle‐Ottawa Scale.

**Results:**

A total of 785 participants across 11 studies were included, with 387 allocated to bracing and 388 to non‐bracing groups. Meta‐analysis revealed no significant differences between groups in IKDC objective score (odds ratio [OR] = 1.18; 95% confidence interval [CI], 0.65–2.14; *p* = 0.58), Lysholm score (mean difference [MD] = −0.30; 95% CI, −0.72 to 0.11; *p* = 0.15), Tegner score (MD = −0.22; 95% CI, −0.46 to 0.02; *p* = 0.07), VAS pain score (MD = 0.08; 95% CI, −0.15 to 0.32; *p* = 0.49), single‐leg hop test (MD = 1.06; 95% CI, −0.01 to 2.14; *p* = 0.05), and anterior–posterior knee laxity (MD = −0.30; 95% CI, −0.72 to 0.11; *p* = 0.15). Subgroup analyses indicated significantly better Lysholm and Tegner scores among individuals without bracing when follow‐up exceeded 2 years. No consistent differences were observed by graft type.

**Conclusion:**

Postoperative bracing did not yield significant improvements in function, pain, strength, or stability following ACLR. Mid‐ to long‐term outcomes (follow‐up >2 years, up to 5 years) may favour non‐bracing, indicating that routine brace use after ACLR is not warranted.

**Level of Evidence:**

Level II, systematic review.

**Abbreviations**
ACL: anterior cruciate ligament
ACLR: anterior cruciate ligament reconstruction
AOSSOM: American orthopedic society for sports medicine
BPTB: bone‐patellar tendon‐bone
CI: confidence interval
IKDC: International Knee Documentation Committee
MD: mean difference
NOS: Newcastle‐Ottawa
OR: odds ratio
PRISMA: Preferred Reporting Items for Systematic Reviews and Meta‐Analysis
RCTs: randomized controlled trails
ROM: range of motion
VAS: visual analogue scale

## INTRODUCTION

The anterior cruciate ligament (ACL) is frequently injured in sports and, particularly in young and active individuals, reconstruction remains the standard treatment to restore knee stability and function [7, 30, 36, 38]. Autografts—typically bone–patellar tendon‐bone (BPTB) or hamstring tendons—and, less commonly, allografts are usually employed for ACL reconstruction (ACLR).

Surgical intervention represents only one aspect of comprehensive treatment. A well‐structured postoperative rehabilitation programme is crucial to restore knee function, ensure a safe return to activity, and minimise re‐injury risk [27]. According to the American Orthopaedic Society for Sports Medicine, up to 85% of individuals undergoing ACLR are prescribed postoperative knee bracing [8]. Braces are commonly used for 3 weeks to 3 months postoperatively [2, 4, 11, 15, 37], with proposed benefits such as pain relief, restriction of range of motion, and external knee stabilization [33]. The rationale for bracing is to reduce mechanical stress on the healing graft and facilitate early mobilization by limiting joint stress during the early phase of ligamentisation [1, 2, 34]. Some biomechanical studies suggest that bracing may offload the graft in individuals with quadriceps or hamstring weakness [9, 25, 26]. Other theoretical benefits include improved proprioception, reduced extension deficits, and prevention of tunnel widening [22, 40, 41].

However, the clinical utility of postoperative bracing has been increasingly questioned in recent years. Prolonged brace use may have detrimental effects, such as muscular atrophy and impaired proprioception, due to disuse of the periarticular musculature [5]. Bracing may also cause discomfort and inconvenience to individuals [21]. Several studies have failed to demonstrate any significant benefits of bracing in combination with standard rehabilitation protocols. For example, Brandsson et al. reported that bracing did not influence long‐term outcomes, including knee laxity, at 2 years after ACLR [4]. Similarly, Kartus et al. found no differences in knee stability, functional scores, or complication rates at 2 years between braced and non‐braced individuals during early postoperative rehabilitation [15]. Yang et al. conducted a meta‐analysis which found that knee bracing did not improve clinical outcomes, including function and stability, after ACLR. Moreover, they speculated that bracing might have potential drawbacks, such as adverse effects on knee outcomes and increased healthcare costs [43]. However, the previous meta‐analysis included a limited number of studies and did not perform subgroup analyses, making it challenging to determine the impact of factors such as graft type and follow‐up duration on the findings. To address this gap, the present study incorporated two additional randomized controlled trials and two case‐control studies and further conducted subgroup analyses based on follow‐up duration (≥2 years vs. <2 years) and graft type (semitendinosus with or without gracilis, patellar tendon, and BPTB). A more comprehensive understanding of the influence of bracing on postoperative recovery following ACLR is achieved through this approach.

Given the conflicting evidence, a comprehensive assessment of the effectiveness of postoperative bracing on knee function and stability is required. We conducted this systematic review and meta‐analysis to examine the influence of knee bracing after ACLR on functional recovery, including International Knee Documentation Committee (IKDC) subjective score, Lysholm score, Tegner activity score, pain (visual analogue scale [VAS]), muscle strength (single‐leg hop test), and joint laxity (side‐to‐side difference), to provide evidence‐based recommendations for postoperative rehabilitation strategies in individuals undergoing ACLR.

## METHODS

### Search strategy

The search strategy for this study was conducted in accordance with the guidelines formulated by the Preferred Reporting Items for Systematic Reviews and Meta‐Analyses (PRISMA) [29]. Studies published from the start of each database through March 2025 were systematically searched in PubMed, EMBASE, and the Cochrane Library. The search strategy combined terms related to ACLR, knee bracing, and postoperative rehabilitation. Duplicate records were excluded, and studies were screened independently by two reviewers based on the title, abstract, and full text. Any inconsistencies were reconciled collaboratively, with involvement of a third reviewer if consensus could not be achieved.

### Inclusion and exclusion criteria

Studies were considered for inclusion if they fulfilled the following conditions: (1) randomized controlled trials (RCTs) or retrospective cohort studies; (2) individuals undergoing primary ACLR, with no restrictions on age, level of sports participation, or body mass index (BMI); (3) comparison between postoperative knee bracing and no bracing; (4) reported outcomes on knee function, pain, muscle strength, or joint stability and (5) no language restrictions. The exclusion criteria were as follows: (1) non‐comparative studies; (2) ACL revision surgery; (3) insufficient data and (4) animal or cadaveric studies.

### Data extraction and outcome measures

Data extraction was undertaken independently by the two authors, including study characteristics, sample size, participant demographics, graft type, duration of brace use, and outcome measures, using a standardized form. The primary outcomes were functional scores (IKDC subjective, Lysholm, and Tegner activity scores), pain (VAS), muscle strength (single‐leg hop test), and joint laxity (KT‐1000, measured side‐to‐side difference).

### Quality assessment

Methodological quality assessment for the RCTs was conducted utilizing the Cochrane Collaboration's Risk of Bias tool. Using the Newcastle–Ottawa Scale (NOS), retrospective studies achieving scores of 7 or more were deemed to be of high quality.

### Statistical analysis

The meta‐analysis was implemented with the assistance of Review Manager 5.3. The computation of mean differences (MDs) and corresponding 95% confidence intervals (CIs) was performed for continuous variables. Dichotomous variables were analysed using odds ratios (ORs) and 95% CIs. Between‐study heterogeneity was evaluated using the *I*² statistic and *χ* ^2^ test. A fixed‐effects model was adopted when heterogeneity was considered low (*I*² < 50% and *p* > 0.05); otherwise, a random‐effects model was employed. To explore potential heterogeneity sources, subgroup and sensitivity analyses were performed based on follow‐up duration and graft type. *p*‐Values less than 0.05 indicated statistical significance.

## RESULTS

### Study selection

**Figure 1:**
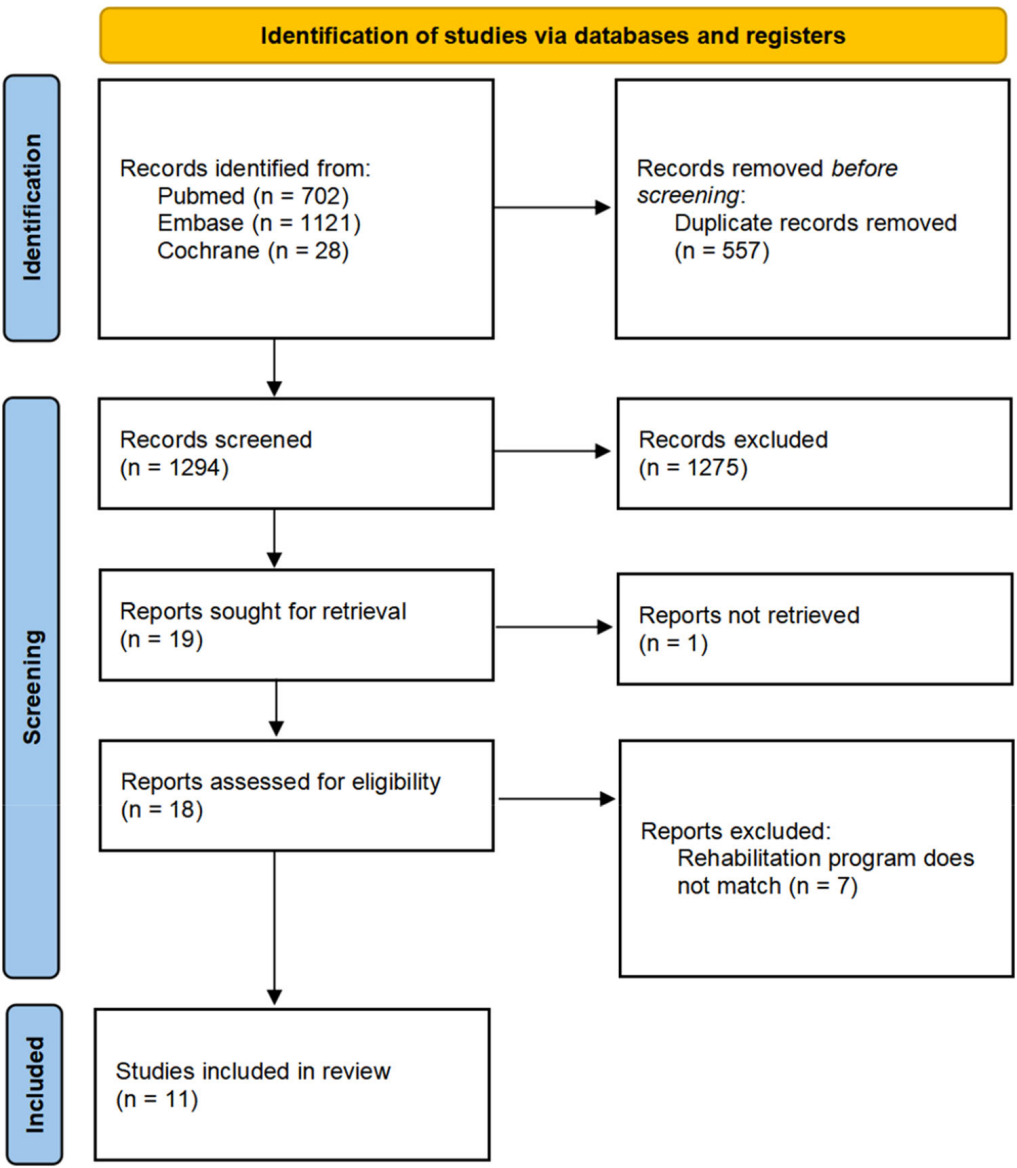
PRISMA (Preferred Reporting Items for Systematic Reviews and Meta‐Analyses) flowchart of study inclusion.

The database search initially yielded 1851 records. Following duplicate removal and title/abstract screening, 19 studies remained eligible for full‐text assessment. Following full‐text review, 11 studies satisfied the eligibility requirements and were included in the final meta‐analysis. Refer to Figure 1 (PRISMA flow diagram) for the study selection workflow (Figure 1).

### Quality assessment

**Figure 2:**
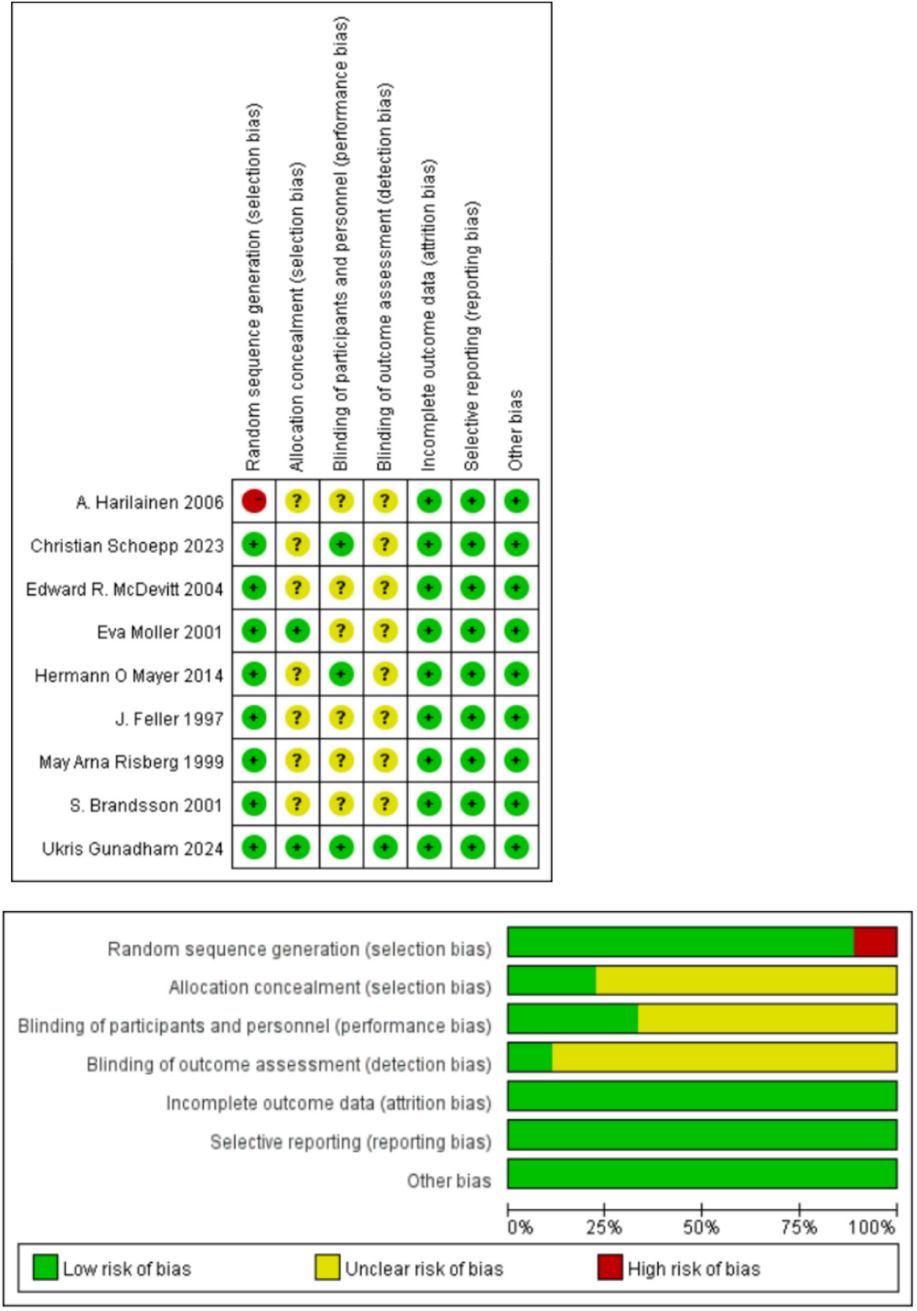
Quality evaluation results of randomized controlled studies.

Among the included studies, nine were RCTs and two were retrospective case‐control studies. All RCTs were rated as having a moderate risk of bias (grade B), based on the Cochrane Risk of Bias Tool (Figure 2). Cohort studies were assessed using the NOS, with scores of 7 and 5, respectively, indicating moderate methodological quality (Table 1).

**Table 1:** Quality evaluation results of case‐control study.

Study ID	Selection				Comparability	Exposure			Score
	1	2	3	4		a	b	c	
Furkan Yapıcı 2022	★	★		★	★	★	★	★	7
Jüri Kartus 1997	★	★				★	★	★	5

*Note*: 1: Is the case definition adequate?; 2: Representativeness of the cases; 3: Selection of Controls; 4: Definition of Controls; a: Ascertainment of exposure; b: Same method of ascertainment for cases and controls; c: Non‐Response rate.

A total of 785 individuals were included, with 387 and 388 individuals in the bracing and non‐bracing groups, respectively. The studies reported comparable baseline characteristics between the groups, including age and sex distribution (*p* > 0.05). An overview of the included studies is provided in Table 2.

**Table 2:** Basic characteristics of included studies.

Study ID	Type of study	Country	Group	Patients (*N*)	Dropped (*N*)	Age (years)	Male (%)	Graft used for ACL reconstruction	Fixation method	Type of brace	Treatment period with brace post‐op	Follow‐up
Ukris Gunadham, 2024 [13]	Randomized controlled trial	Thailand	Brace	42	7	Mean: 30.8 ± 9.6	35	Semitendinosus: 83.3%	F: suspension device T: interference screw	Functional brace	4 weeks	2 years
								Semitendinosus + Gracilis: 16.7%				
			No brace	42	2	Mean: 29.1 ± 9.4	40	Semitendinosus: 85.7%				
								Semitendinosus + Gracilis: 14.3%				
Christian Schoepp, 2023 [35]	Randomized controlled trial	Germany	Brace	68	10	Mean: 33.2 ± 12.3	37	Semitendinosus (or + Gracilis)	F: extracortical button device T: bioabsorbable screw and washer	Functional brace	6 weeks	1 year
			No brace	70	14	Mean: 31.5 ± 10.8	37					
Furkan Yapıcı, 2022 [44]	Case control study	Türkiye	Brace	56	0	Mean: 31.7 ± 8.8	94.6	Semitendinosus (or + Gracilis)	F: endobutton fixationT: bioabsorbable screw and staple fixation	Rehabilitative brace	3 weeks	Mean: 40.2 ± 9.4 months
			No brace	63	0	Mean: 32.2 ± 8.5	93.7					Mean: 36.9 ± 8.7 months
Mayr, 2014 [20]	Randomized controlled trial	Germany	Brace	27	5	Mean: 40 ± 11	81.5	BPTB‐auto	F&T: titanium interference screw	Functional brace	6 weeks	4 years
			No brace	25	5	Mean: 35 ± 8	88.0					
Harilainen, 2006 [14]	Randomized controlled trial	Finland	Brace	23	7	Median: 26 (16–42)	53.3	PT‐auto	F&T: metal interference screw	Rehabilitative brace	12 weeks	Median: 5 (4.7–6.2) years
			No brace	25	5	Median: 25 (15–50)	60.0					Median: 5 (3.6–6) years
McDevitt, 2004 [21]	Randomized controlled trial	USA	Brace	47	3	N/A	N/A	BPTB‐auto	F&T: interference metallic screw	Functional brace	6 weeks	Median: 29 (24–42) months
			No brace	48	2							
Möller, 2001 [24]	Randomized controlled trial	Sweden	Brace	27	3	Median: 28 (21–53)	50.0	PT‐auto	F&T: titanium interference screw	Rehabilitative brace	6 weeks	2 years
			No brace	29	3	Median: 31 (19–48)	46.9					
Brandsson, 2000 [4]	Randomized controlled trial	Sweden	Brace	23	2	Median: 28.5 (15–42)	76.0	BPTB‐auto	F&T: interference screws	Rehabilitative brace	3 weeks	Median: 26 (24–27) months
			No brace	20	5	Median: 25 (16–40)	72.0					Median: 29 (24–42) months
Risberg, 1999 [32]	Randomized controlled trial	Norway	Brace	28	2	Mean: 28 (15–47)	53.0	BPTB‐auto	N/A	Rehabilitative brace	12 weeks	2 years
			No brace	28	2							
Jüri Kartus, 1997 [15]	Case control study	Sweden	Brace	39	0	Median: 27 (16–48)	71.8	PT‐auto	F&T: interference screws	Rehabilitative brace	4 (3–6) weeks	Median: 25 (23–28) months
			No brace	39	0	Median: 26 (14–51)	59.0					Median: 26 (25–27) months
Feller, 1997 [10]	Randomized controlled trial	Australia	Brace	19	1	Mean: 30 ± 9	60.0	PT‐auto	F&T: interference screws	Rehabilitative brace	6 weeks	4 months
			No brace	20	0	Mean: 28 ± 9	60.0					

Abbreviations: ACL, anterior cruciate ligament; BPTB‐auto, bone‐patella tendon‐bone autograft; F, femoral; PT‐auto, patella tendon autograft; T, tibial.

### Meta‐analysis outcomes

**Figure 3:**
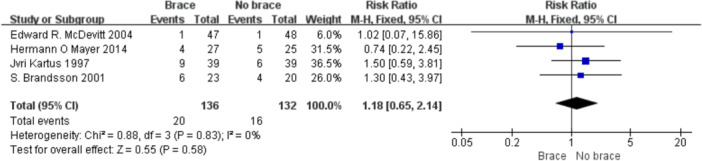
Forest plot comparing IKDC objective scores with and without braces after anterior cruciate ligament reconstruction.

**Figure 4:**
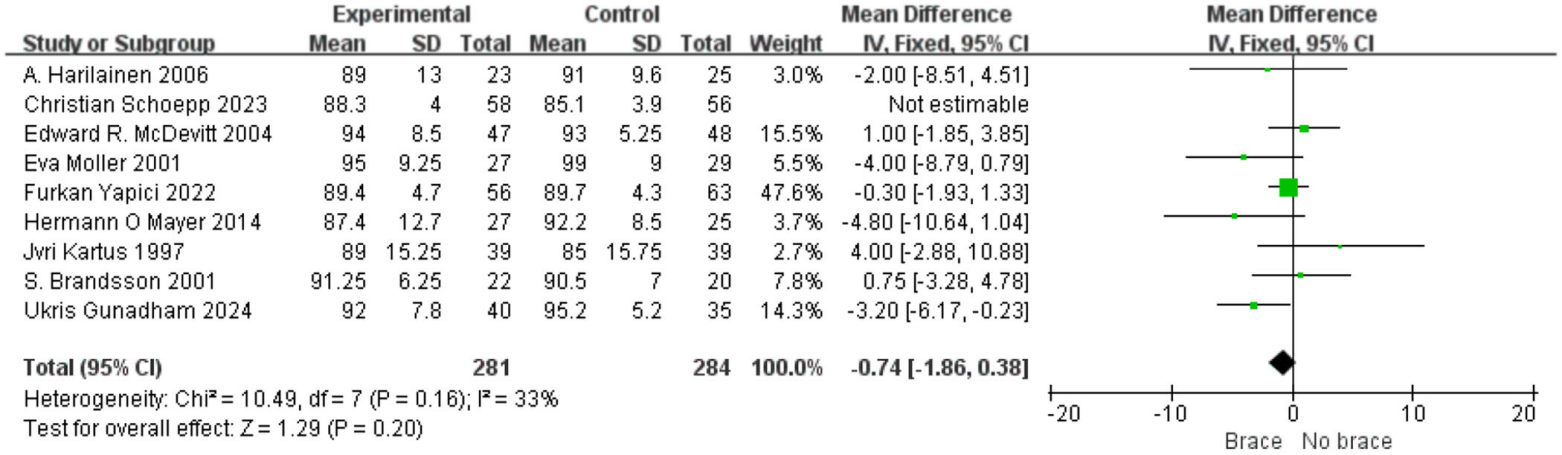
Forest plot comparing Lysholm scores with and without braces after anterior cruciate ligament reconstruction. CI, confidence interval; SD, standard deviation.

**Figure 5:**
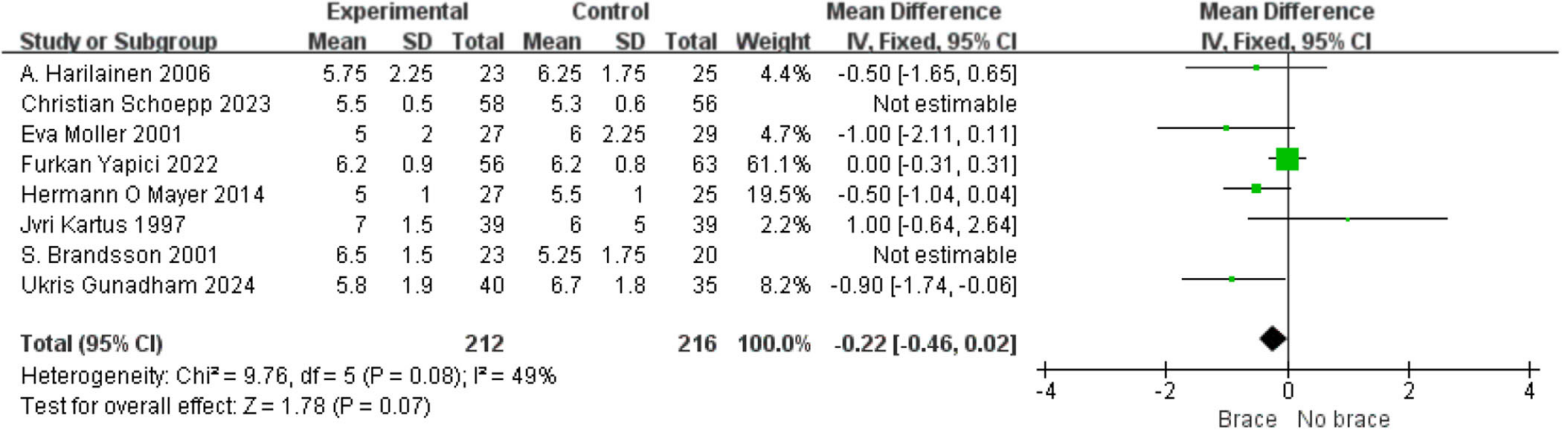
Forest plot comparing Tegner scores with and without braces after anterior cruciate ligament reconstruction. CI, confidence interval; SD, standard deviation.

**Figure 6:**
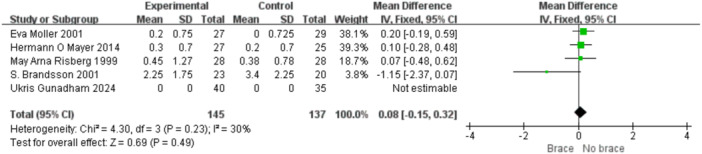
Forest plot comparing VAS pain scores with and without braces after anterior cruciate ligament reconstruction. CI, confidence interval; SD, standard deviation.

**Figure 7:**
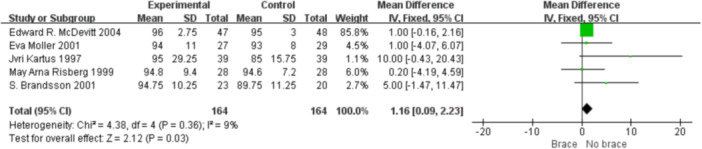
Forest plot comparing single‐leg hop test with and without braces after anterior cruciate ligament reconstruction. CI, confidence interval; SD, standard deviation.

**Figure 8:**
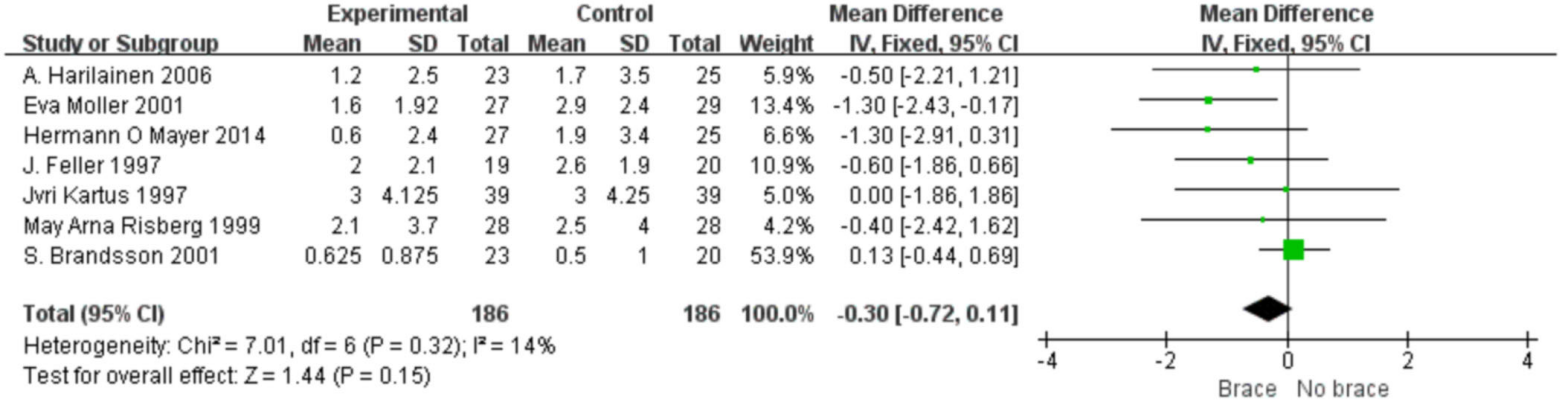
Forest plot comparing side‐to‐side difference with and without braces after anterior cruciate ligament reconstruction. CI, confidence interval; SD, standard deviation.

1. IKDC subjective score
  Four studies reported the postoperative IKDC subjective scores. The proportion of individuals who achieved grade A (normal) was 14.71% (20/136) in the bracing group and 12.12% (16/132) in the non‐bracing group. A fixed‐effects model was used, and the pooled analysis showed no significant difference between the groups (OR = 1.18; 95% CI, 0.65– 2.14; *p* = 0.58), indicating no advantage of bracing in terms of subjective knee function (Figure 3).
2. Lysholm score
  Nine studies reported the Lysholm scores. Using a random‐effects model, no significant difference was observed between the bracing and non‐bracing groups (MD = −0.31; 95% CI, −2.31 to 1.69; *p* = 0.76). After removing one outlier, the heterogeneity decreased (*p* = 0.16, *I*² = 33%). A fixed‐effects model again revealed no significant difference (MD = −0.93; 95% CI, −2.53 to 0.66; *p* = 0.20) (Figure 4).
3. Tegner activity score
  Eight studies reported Tegner scores. A random‐effects model found no significant difference between the groups (MD = 0.09; 95% CI, −0.47 to 0.29; *p* = 0.63). After excluding two studies, heterogeneity was reduced (*p* = 0.08, *I*² = 49%), and fixed‐effects analysis still found no significant difference (MD = −0.22; 95% CI, −0.46 to 0.02; *p* = 0.07) (Figure 5).
4. VAS pain score
  Five studies reported postoperative VAS pain scores. A a fixed‐effects model was applied and the pooled analysis showed no significant difference between the bracing and non‐bracing groups (MD = 0.08; 95% CI, −0.15 to 0.32; *p* = 0.49), indicating that postoperative bracing had no impact on perceived pain levels (Figure 6).
5. Single‐leg hop test
  Five studies evaluated postoperative muscle strength using the single‐leg hop test. A fixed‐effects model was used and the pooled results initially suggested a significant advantage in the bracing group (MD = 1.16; 95% CI, 0.09 to 2.23; *p* = 0.03). However, after excluding one low‐quality case‐control study, the result was no longer statistically significant (MD = 1.06; 95% CI, −0.01 to 2.14; *p* = 0.05), suggesting that bracing does not provide a consistent advantage in muscle strength recovery (Figure 7).
6. Side‐to‐side difference (KT‐1000 measurement)
  Seven studies reported anterior–posterior knee laxity using the KT‐1000 device. Fixed‐effects analysis showed no significant difference between groups [MD = −0.30; 95% CI, −0.72 to 0.11; *p* = 0.15], indicating no measurable improvement in mechanical stability with brace use (Figure 8).

### Subgroup analyses

**Figure 9:**
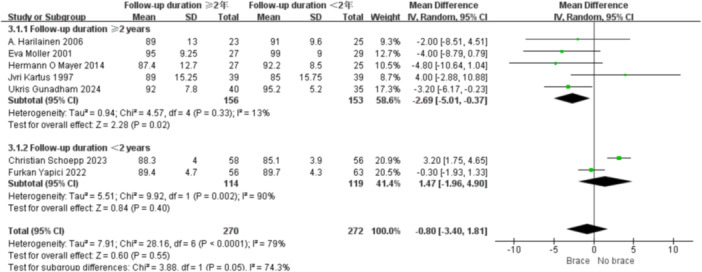
Forest plot comparing the Lysholm scores between the two groups: Follow‐up time of ≥2 years and <2 years. CI, confidence interval; SD, standard deviation.

**Figure 10:**
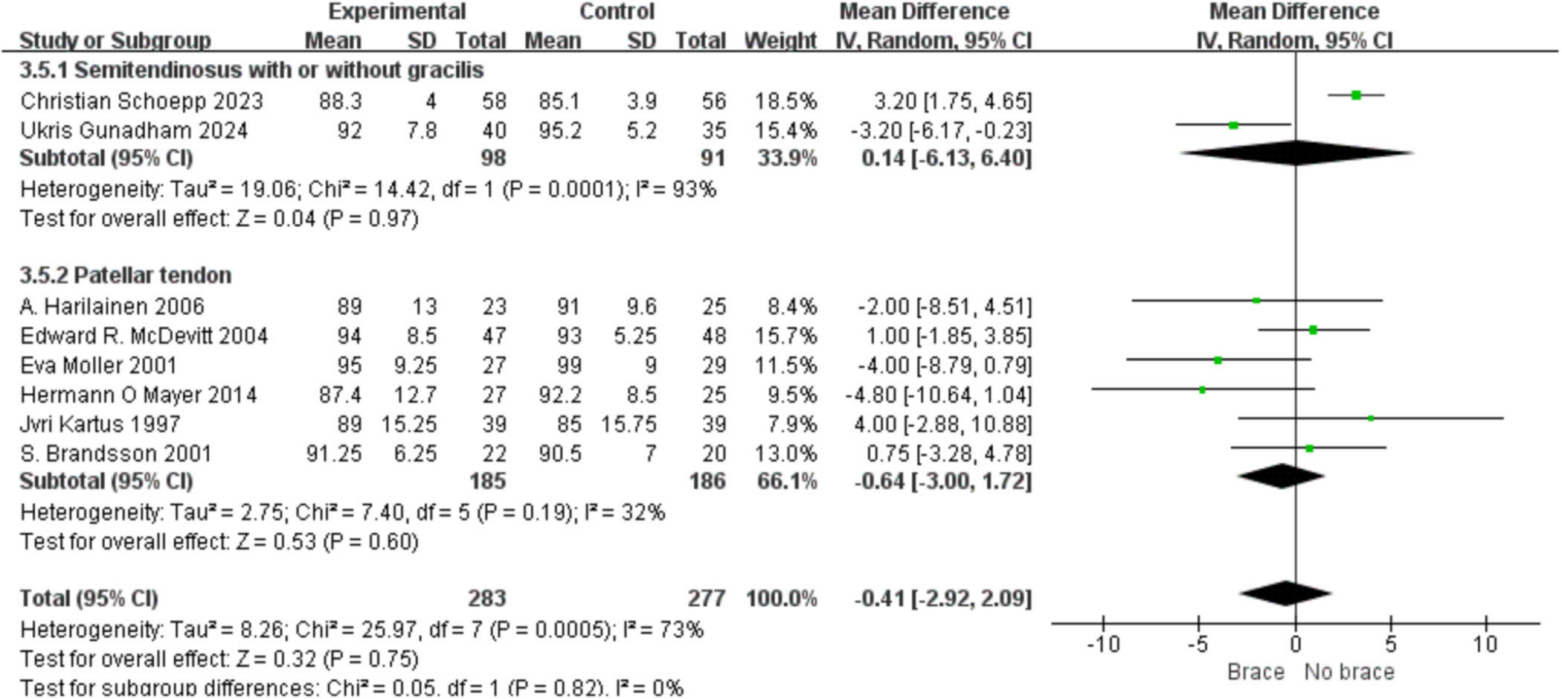
Forest plot comparing the Lysholm scores between the two groups: The graft types are semitendinosus (or + gracilis) and PT‐auto. CI, confidence interval; SD, standard deviation.

**Figure 11:**
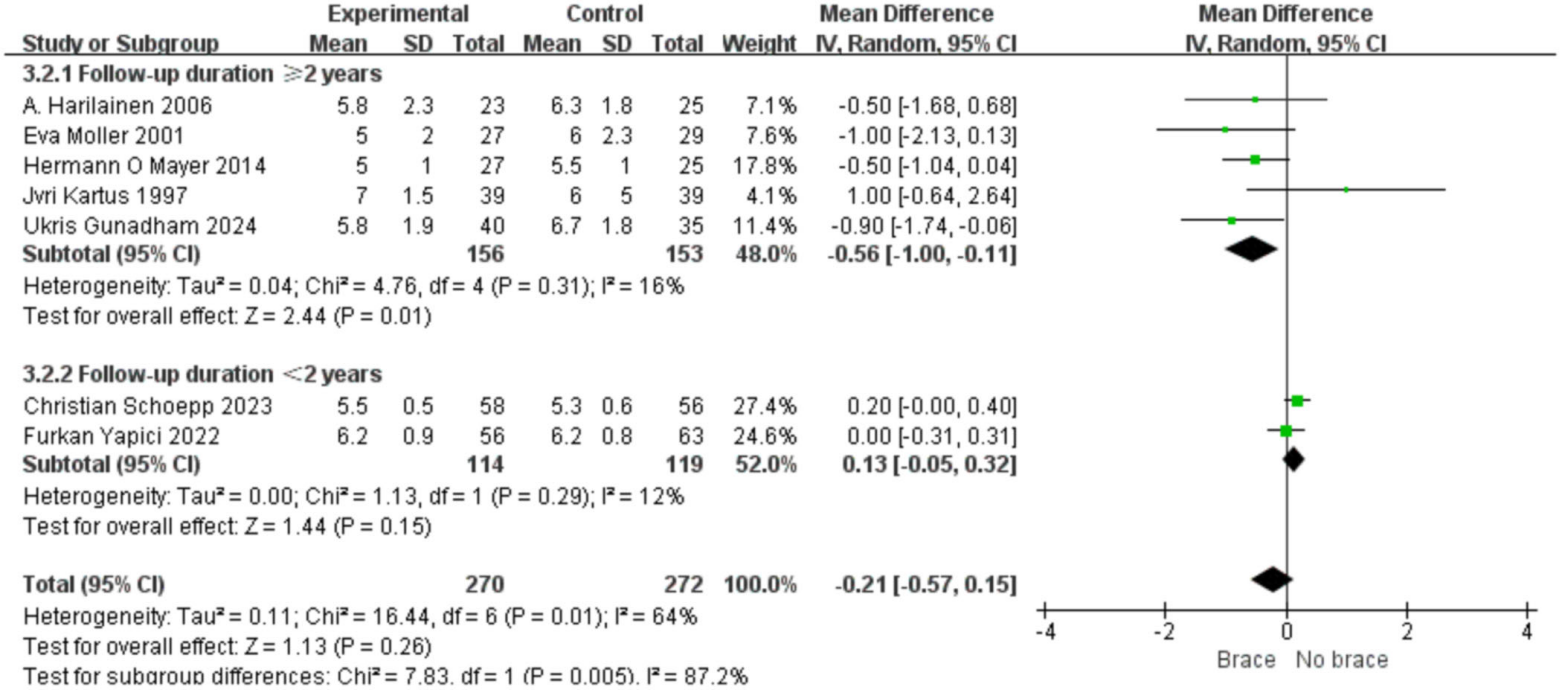
Forest plot comparing the Tegner scores between the two groups: Follow‐up time of ≥2 years and <2 years. CI, confidence interval; SD, standard deviation.

**Figure 12:**
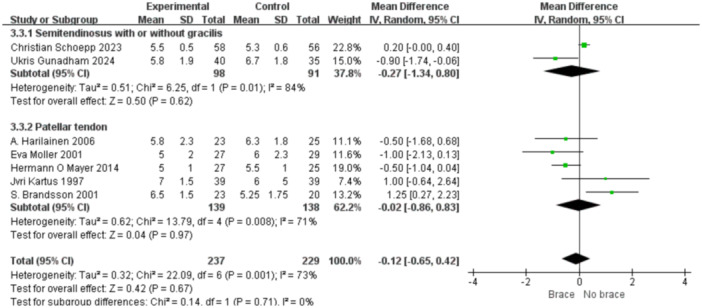
Forest plot comparing the Lysholm scores between the two groups: The graft types are semitendinosus (or + gracilis) and PT‐auto. CI, confidence interval; SD, standard deviation.

1. Lysholm score
  By follow‐up duration:
  Subgrouping by follow‐up duration (≥2 years vs. <2 years) revealed high overall heterogeneity (*I*² = 79%, *p* < 0.0001). No statistically significant difference was observed in the overall comparison (MD = −0.80; 95% CI, −3.40 to 1.81; *p* = 0.55). In studies with ≥2 years follow‐up, heterogeneity decreased (*I*² = 37%, *p* = 0.16), and the non‐bracing group had significantly higher scores (MD = −2.69; 95% CI, −5.01 to −0.37; *p *= 0.02) (Figure 9).
  By graft type:
  Subgrouping according to graft type (hamstring vs. patellar tendon) also showed high heterogeneity (*I*² = 73%, *p* = 0.0005). No overall difference was found (MD = −0.41; 95% CI, −2.92 to 2.09; *p* = 0.75). In the patellar tendon group, bracing showed a trend toward lower scores (MD = −0.64; 95% CI, −3.00 to 1.72; *p *= 0.60), but this was not statistically significant (Figure 10).
2. Tegner score
  By follow‐up duration:
  Subgroup analysis showed that in studies with ≥2 years follow‐up, the bracing group had significantly lower scores (MD = −0.36; 95% CI, −0.80 to −0.07; *p* = 0.01), with minimal heterogeneity (*I*² = 16%, *p *= 0.31). In shorter follow‐up studies, No statistically significant differences were observed (Figure 11).
  By graft type:
  In both the hamstring and patellar tendon graft subgroups, intergroup differences were not statistically significant, while heterogeneity remained substantial (*I*² > 50%) (Figure 12).
3. Side‐to‐side difference (KT‐1000 measurement)

**Figure 13:**
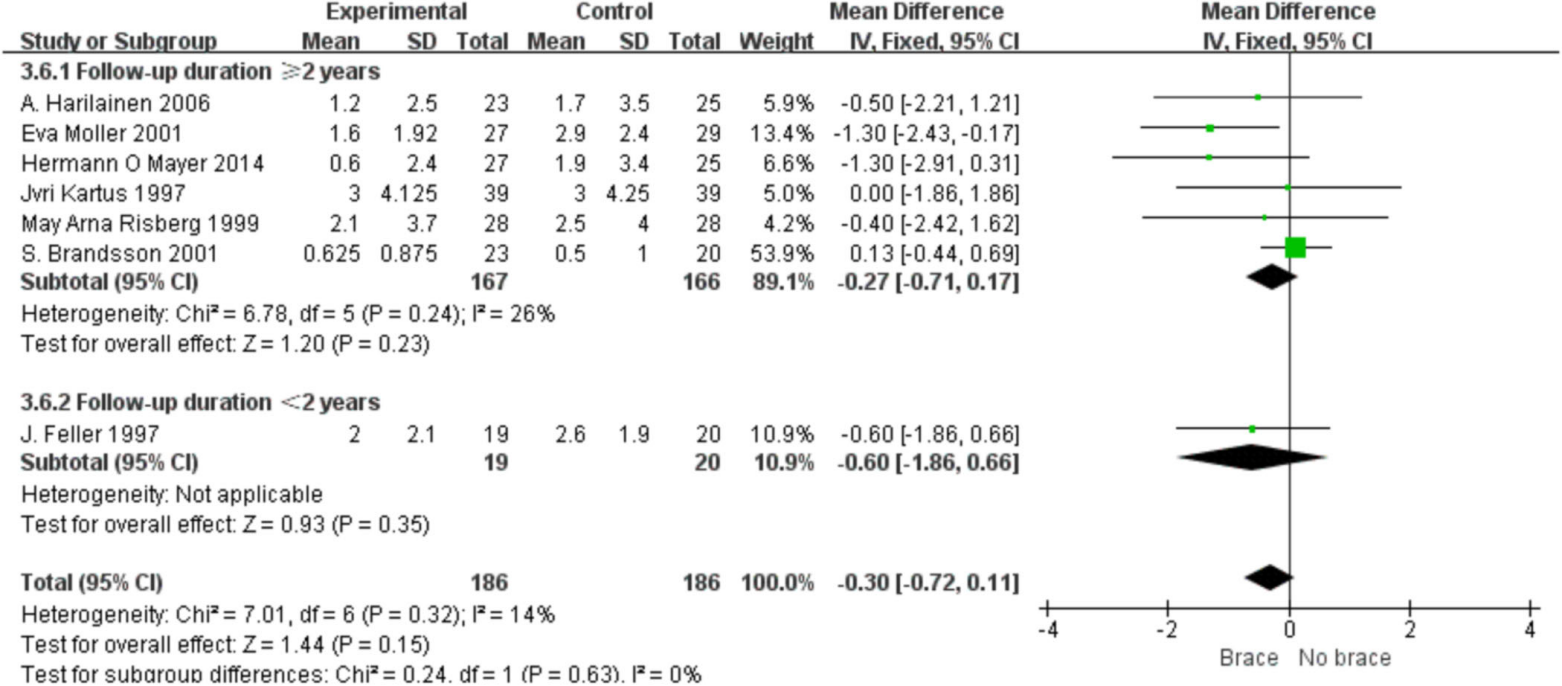
Forest plot comparing the side‐to‐side difference between the two groups: follow‐up time of ≥2 and <2 years. CI, confidence interval; SD, standard deviation.

By follow‐up duration:

No statistically significant difference was observed in either the short‐ or long‐term follow‐up subgroups. (Figure 13).

**Figure 14:**
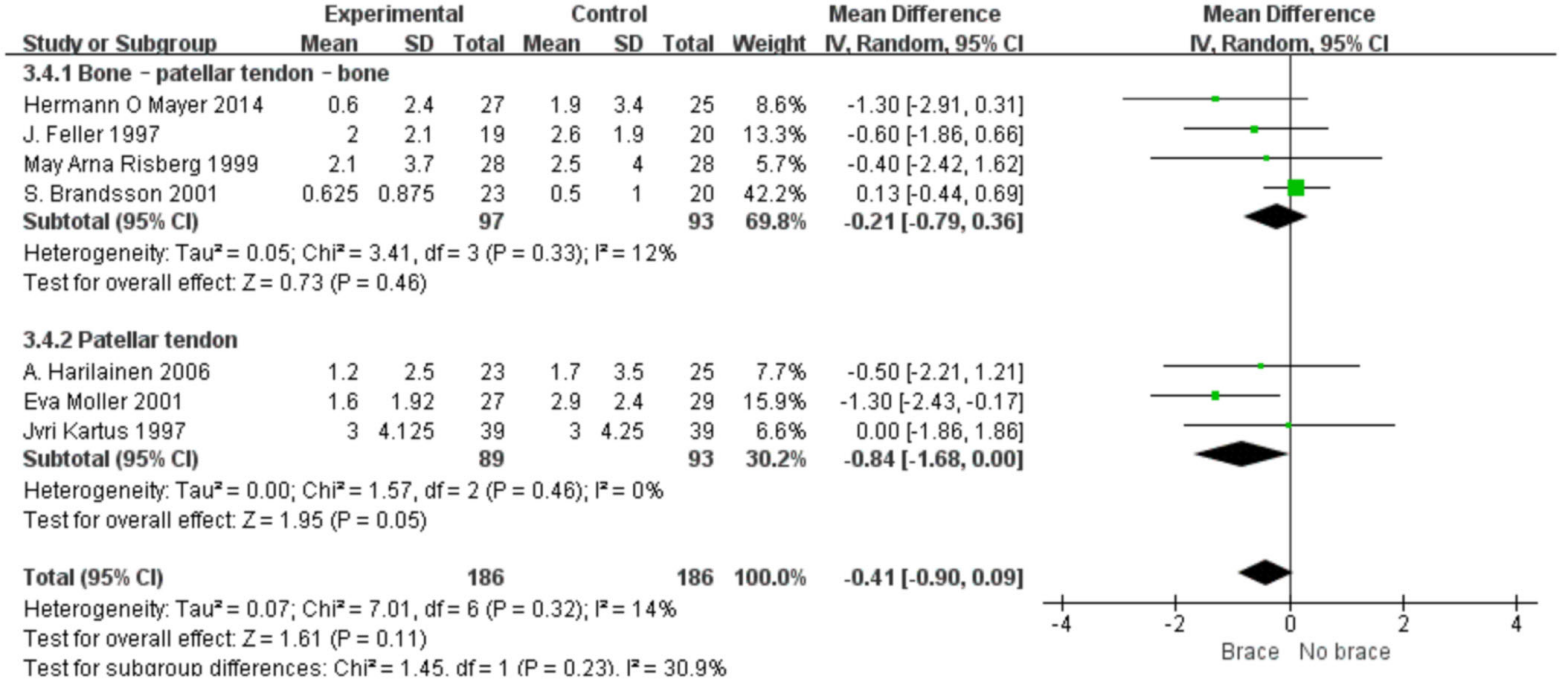
Forest plot comparing the Lysholm scores between the two groups: The graft types are BPTB‐auto and PT‐auto. CI, confidence interval; SD, standard deviation.

By graft type:

Subgroup analysis revealed no significant difference for either bone–patellar tendon‐bone or hamstring grafts, though the patellar tendon subgroup showed a non‐significant trend favouring the non‐bracing group (MD = −0.84; 95% CI, −1.68 to 0.00; *p* = 0.05) (Figure 14).

## DISCUSSION

The most important finding of this study was that postoperative knee bracing did not significantly improve functional scores (IKDC subjective, Lysholm, and Tegner activity scores), pain control (VAS), muscle strength (single‐leg hop test), or mechanical stability (KT‐1000‐measured laxity), suggesting that routine bracing after ACLR may not be necessary for optimal recovery.

Although ACLR is well‐established for restoring knee function in young and physically active individuals, the role of postoperative bracing remains controversial. Proponents argue that bracing reduces mechanical stress on the healing graft, mitigates quadriceps inhibition, and provides proprioceptive support during the early phases of rehabilitation. Some studies have reported that bracing may reduce tunnel widening in the early postoperative period, potentially preserving graft integrity. For instance, Vadalà et al. noted that full extension bracing during the first 2 weeks after surgery might limit tunnel expansion [41]. Pezzullo et al. suggested that while no strong evidence supports bracing for preventing re‐injury (except in skiing), braces may help athletes regain confidence during return‐to‐sport [31].

However, an increasing body of evidence challenges these assumptions. Bracing may restrict joint mobility, leading to muscle atrophy and impaired proprioception. Several randomized trials have shown no significant benefits of bracing over standard rehabilitation protocols [12]. Christensen et al. found no differences in functional outcomes or anterior laxity at 12 and 24 weeks postoperatively [6]. Lindström et al. similarly reported that bracing for 3 weeks post‐ACLR had no effect on 1‐year outcomes, including range of motion, subjective scores, strength, and effusion [17, 18]. Mayr et al. observed no differences in the Lysholm, Tegner, or laxity scores between the braced and non‐braced groups over 2–5 years [20]. Multiple systematic reviews and meta‐analyses—including those by Rodríguez‐Merchán, Kruse, Smith, Wright and Masini, have echoed these findings, concluding that knee braces offer no substantial benefit in terms of pain relief, stability, or long‐term functional recovery [16, 19, 33, 37, 42]. Some researchers have also suggested that prolonged bracing can weaken the vastus medialis obliquus muscle and potentially compromise joint function [5, 28, 39].

Although one outlier study appeared to show a benefit in single‐leg hop test performance, this was attributed to the inclusion of a low‐quality case‐control study. When excluded, this advantage disappeared, aligning our results with those of the rest of the literature. The overall findings may reflect the high mechanical stability afforded by modern arthroscopic techniques and graft fixation methods, as well as the efficacy of accelerated rehabilitation protocols.

Subgroup analyses revealed that in studies with ≥2 years of follow‐up, the non‐bracing group performed better on Lysholm and Tegner scores, although statistical significance varied. This trend suggests that long‐term functional adaptation and neuromuscular re‐education are not compromised by omitting bracing. However, the underlying mechanisms require further investigation.

Analyses according to graft type revealed no consistent interactions between graft selection and bracing efficacy. Although minor variations were observed (e.g., slightly better Lysholm scores with hamstring grafts in the bracing group and slightly better Tegner scores with patellar tendon grafts in the non‐bracing group), none reached statistical significance. Our findings are consistent with prior work by Mohtadi et al., Barber‐Westin, and Zaffagnini, who found no clear differences in return‐to‐sport timelines or clinical outcomes based on graft type [3, 23, 45].

The lack of significant differences between the bracing and non‐bracing groups, despite the theoretical mechanical and proprioceptive advantages of orthotic fitting, may be explained by several factors. First, modern arthroscopic techniques and advanced graft fixation devices (e.g., interference screws, cortical buttons) provide high initial stability, which likely reduces the marginal benefit of additional external support. Second, the widespread adoption of accelerated rehabilitation protocols, emphasizing early range of motion and weight‐bearing, may counteract the potential restrictive effects of bracing and promote functional recovery without the need for immobilization. Furthermore, although surgeon bias could influence the decision to use an orthosis, its impact is likely minimal. Many studies in our analysis used blinded outcome assessments, randomization, and standardized rehabilitation protocols, which helped reduce bias in treatment allocation and outcome evaluation. While individual surgeon judgement may still play a role, these measures significantly minimized its effect on the results.

Meanwhile, the role of different rehabilitation protocols and the duration of orthotic fitting may play significant roles in influencing postoperative outcomes. Early rehabilitation, particularly protocols emphasizing early mobilization and progressive loading, may reduce the need for external bracing by promoting muscle strength and joint mobility. On the other hand, the duration of bracing could have contrasting effects. Short‐term bracing in the immediate postoperative phase may provide psychological reassurance and stability, whereas prolonged use might restrict joint mobility, leading to muscle atrophy and impaired proprioception. This could hinder neuromuscular control and slow functional recovery, especially in protocols emphasizing early range of motion. Therefore, the balance between bracing duration and rehabilitation intensity is crucial for optimizing recovery outcomes.

Despite the subgroup and sensitivity analyses, high heterogeneity persisted in the Lysholm (*I*² = 72%) and Tegner (*I*² = 69%) scores. Potential sources of bias include variability in postoperative rehabilitation protocols, inconsistent brace duration, and differing follow‐up periods. Short‐ and long‐term follow‐up studies yielded divergent results; however, subgroup analyses did not fully account for this heterogeneity.

## LIMITATIONS

This meta‐analysis has some limitations. First, the inclusion of one low‐quality case‐control study may introduce bias, although it contributed to the sample size, and its influence was minimized through sensitivity analysis and the use of random‐effects models. Second, since there were few related studies with follow‐up times of <2 years, there was no significant difference between the two subgroups in the analysis of subgroups based on follow‐up time. Finally, Due to the limited number of available studies, subgroup analyses based on surgical approach (arthroscopic vs. open), graft type (autograft vs. synthetic), and rehabilitation protocol could not be performed. Future large‐scale multicentre RCTs are required to address these questions.

## CONCLUSION

Based on current evidence, the routine use of knee bracing following ACLR does not confer significant benefits in terms of functional outcomes, joint stability, or pain control. However, subgroup analyses suggest potential long‐term advantages in functional outcomes for patients not using braces, particularly those followed for ≥2 years. Clinical decision‐making should be individualized, with greater emphasis on optimizing surgical techniques and rehabilitation protocols. Additional well‐designed studies are required to clarify the potential benefits of bracing in distinct patient subgroups. Future studies should consider a differentiated approach, particularly focusing on athletes and non‐athletes, as the demands on knee stability and functional recovery may differ significantly between these groups.

## AUTHOR CONTRIBUTIONS

All authors contributed to the study conception and design. Qitai Lin, Xueding Wang and Zehao Li contributed to the conception and design of the study. Qitai Lin, Xueding Wang and Zehao Li contributed to the acquisition of the data. Qitai Lin, Qian Li, Wenming Yang and Yugang Xing conducted the statistical analysis. Wangping Duan, and Pengcui Li contributed to the interpretation of the data. The first draft of the manuscript was written by Qitai Lin and all authors commented on previous versions of the manuscript. All authors read and approved the final manuscript.

## CONFLICT OF INTEREST STATEMENT

The authors declare no conflicts of interest.

## ETHICS STATEMENT

The authors have nothing to report.

## Data Availability

The data that support the findings of this study are available from the corresponding author upon reasonable request.

## References

[1] Amiel D , Kleiner JB , Akeson WH . The natural history of the anterior cruciate ligament autograft of patellar tendon origin. Am J Sports Med. 1986;14:449–462. 3799871 10.1177/036354658601400603

[2] Andersson D , Samuelsson K , Karlsson J . Treatment of anterior cruciate ligament injuries with special reference to surgical technique and rehabilitation: an assessment of randomized controlled trials. Arthroscopy. 2009;25:653–685. 19501297 10.1016/j.arthro.2009.04.066

[3] Barber‐Westin SD , Noyes FR . Factors used to determine return to unrestricted sports activities after anterior cruciate ligament reconstruction. Arthroscopy. 2011;27:1697–1705. 22137326 10.1016/j.arthro.2011.09.009

[4] Brandsson S , Faxén E , Kartus J , Eriksson BI , Karlsson J . Is a knee brace advantageous after anterior cruciate ligament surgery? A prospective, randomised study with a two‐year follow‐up. Scand J Med Sci Sports. 2001;11:110–114. 11252459 10.1034/j.1600-0838.2001.011002110.x

[5] Choi EH , Kim KK , Jun AY , Choi EH , Choi SW , Shin KY . Effects of the off‐loading brace on the activation of femoral muscles—a preliminary study. Ann Rehabil Med. 2011;35:887–896. 22506219 10.5535/arm.2011.35.6.887 PMC3309369

[6] Christensen JC , Goldfine LR , West HS . The effects of early aggressive rehabilitation on outcomes after anterior cruciate ligament reconstruction using autologous hamstring tendon: a randomized clinical trial. J Sport Rehabil. 2013;22:191–201. 23579334 10.1123/jsr.22.3.191

[7] Mader K , Pennig D , Dargel J , Gotter M , Koebke J , Schmidt‐Wiethoff R . Biomechanics of the anterior cruciate ligament and implications for surgical reconstruction. Strategies Trauma Limb Reconstr. 2007;2:1–12. 18427909 10.1007/s11751-007-0016-6 PMC2321720

[8] Delay BS , Smolinski RJ , Wind WM , Bowman DS . Current practices and opinions in ACL reconstruction and rehabilitation: results of a survey of the American Orthopaedic Society for Sports Medicine. Am J Knee Surg. 2001;14:85–91. 11401175

[9] Draganich LF , Vahey JW . An in vitro study of anterior cruciate ligament strain induced by quadriceps and hamstrings forces. J Orthop Res. 1990;8:57–63. 2293634 10.1002/jor.1100080107

[10] Feller J , Bartlett J , Chapman S , Delahunt M . Use of an extension‐assisting brace following anterior cruciate ligament reconstruction. Knee Surg Sports Traumatol Arthrosc. 1997;5:6–9. 9127846 10.1007/s001670050016

[11] Feller JA , Cooper R , Webster KE . Current Australian trends in rehabilitation following anterior cruciate ligament reconstruction. Knee. 2002;9:121–126. 11950575 10.1016/s0968-0160(02)00009-1

[12] Glattke KE , Tummala SV , Chhabra A . Anterior cruciate ligament reconstruction recovery and rehabilitation: a systematic review. J Bone Jt Surg. 2022;104:739–754. 10.2106/JBJS.21.00688 34932514

[13] Gunadham U , Woratanarat P . Effect of knee bracing on clinical outcomes following anterior cruciate ligament reconstruction: a prospective randomised controlled study. Asia Pac J Sports Med Arthrosc Rehabil Technol. 2024;36:18–23. 38406661 10.1016/j.asmart.2024.01.006 PMC10891282

[14] Harilainen A , Sandelin J . Post‐operative use of knee brace in bone‐tendon‐bone patellar tendon anterior cruciate ligament reconstruction: 5‐year follow‐up results of a randomized prospective study. Scand J Med Sci Sports. 2006;16:14–18. 16430676 10.1111/j.1600-0838.2004.00435.x

[15] Kartus J , Stener S , Köhler K , Sernert N , Eriksson BI , Karlsson J . Is bracing after anterior cruciate ligament reconstruction necessary? A 2‐year follow‐up of 78 consecutive patients rehabilitated with or without a brace. Knee Surg Sports Traumatol Arthrosc. 1997;5:157–161. 9335027 10.1007/s001670050044

[16] Kruse LM , Gray B , Wright RW . Rehabilitation after anterior cruciate ligament reconstruction: a systematic review. J Bone Jt Surg. 2012;94:1737–1748. 10.2106/JBJS.K.01246 PMC3448301 23032584

[17] Lindström M , Strandberg S , Wredmark T , Felländer‐Tsai L , Henriksson M . Functional and muscle morphometric effects of ACL reconstruction. A prospective CT study with 1 year follow‐up. Scand J Med Sci Sports. 2013;23:431–442. 22107159 10.1111/j.1600-0838.2011.01417.x

[18] Lindström M , Wredmark T , Wretling ML , Henriksson M , Felländer‐Tsai L . Post‐operative bracing after ACL reconstruction has no effect on knee joint effusion. A prospective, randomized study. Knee. 2015;22:559–564. 26051483 10.1016/j.knee.2015.04.015

[19] Masini BD , Owens BD . Current recommendations for anterior cruciate ligament bracing: when to use. Phys Sportsmed. 2013;41:35–39. 23445858 10.3810/psm.2013.02.1997

[20] Mayr HO , Stüeken P , Münch EO , Wolter M , Bernstein A , Suedkamp NP , et al. Brace or no‐brace after ACL graft? Four‐year results of a prospective clinical trial. Knee Surg Sports Traumatol Arthrosc. 2014;22:1156–1162. 23807029 10.1007/s00167-013-2564-2

[21] McDevitt ER , Taylor DC , Miller MD , Gerber JP , Ziemke G , Hinkin D , et al. Functional bracing after anterior cruciate ligament reconstruction: a prospective, randomized, multicenter study. Am J Sports Med. 2004;32:1887–1892. 15572317 10.1177/0363546504265998

[22] Mikkelsen C , Cerulli G , Lorenzini M , Bergstrand G , Werner S . Can a post‐operative brace in slight hyperextension prevent extension deficit after anterior cruciate ligament reconstruction? A prospective randomised study. Knee Surg Sports Traumatol Arthrosc. 2003;11:318–321. 12897981 10.1007/s00167-003-0406-3

[23] Mohtadi NG , Chan DS , Dainty KN , Whelan DB . Patellar tendon versus hamstring tendon autograft for anterior cruciate ligament rupture in adults. Cochrane Database Syst Rev. 2011;9:Cd005960. 10.1002/14651858.CD005960.pub2 PMC6465162 21901700

[24] Möller E , Forssblad M , Hansson L , Wange P , Weidenhielm L . Bracing versus nonbracing in rehabilitation after anterior cruciate ligament reconstruction: a randomized prospective study with 2‐year follow‐up. Knee Surg Sports Traumatol Arthrosc. 2001;9:102–108. 11354851 10.1007/s001670000192

[25] More RC , Karras BT , Neiman R , Fritschy D , Woo SLY , Daniel DM . Hamstrings‐‐an anterior cruciate ligament protagonist. An in vitro study. Am J Sports Med. 1993;21:231–237. 8465918 10.1177/036354659302100212

[26] Naik AA , Das B , Kamat YD . Avoid post operative bracing to reduce ACL rerupture rates. Eur J Orthop Surg Traumatol. 2019;29:1743–1747. 31350649 10.1007/s00590-019-02521-4

[27] Nelson C , Rajan L , Day J , Hinton R , Bodendorfer BM . Postoperative rehabilitation of anterior cruciate ligament reconstruction: a systematic review. Sports Med Arthrosc. 2021;29:63–80. 33972483 10.1097/JSA.0000000000000314

[28] Norozian B , Arabi S , Marashipour SM , Khademi Kalantari K , Akbarzadeh Baghban A , Kazemi SM , et al. Recovery of quadriceps strength and knee function using adjuvant EMG‐BF after primary ACL reconstruction. J Lasers Med Sci. 2023;14:e6. 37089769 10.34172/jlms.2023.06 PMC10114001

[29] Page MJ , McKenzie JE , Bossuyt PM , Boutron I , Hoffmann TC , Mulrow CD , et al. The PRISMA 2020 statement: an updated guideline for reporting systematic reviews. BMJ. 2021;372:n71. 33782057 10.1136/bmj.n71 PMC8005924

[30] Paschos NK , Howell SM . Anterior cruciate ligament reconstruction: principles of treatment. EFORT Open Rev. 2016;1:398–408. 28461919 10.1302/2058-5241.1.160032 PMC5367541

[31] Pezzullo DJ , Fadale P . Current controversies in rehabilitation after anterior cruciate ligament reconstruction. Sports Med Arthrosc. 2010;18:43–47. 20160630 10.1097/JSA.0b013e3181cdb5d3

[32] Risberg MA , Holm I , Steen H , Eriksson J , Ekeland A . The effect of knee bracing after anterior cruciate ligament reconstruction. Am J Sports Med. 1999;27:76–83. 9934423 10.1177/03635465990270012101

[33] Rodríguez‐Merchán EC . Knee bracing after anterior cruciate ligament reconstruction. Orthopedics. 2016;39:e602–e609. 27203412 10.3928/01477447-20160513-04

[34] Rougraff B , Shelbourne KD , Gerth PK , Warner J . Arthroscopic and histologic analysis of human patellar tendon autografts used for anterior cruciate ligament reconstruction. Am J Sports Med. 1993;21:277–284. 8465925 10.1177/036354659302100219

[35] Schoepp C , Ohmann T , Martin W , Praetorius A , Seelmann C , Dudda M , et al. Brace‐free rehabilitation after isolated anterior cruciate ligament reconstruction with hamstring tendon autograft is not inferior to brace‐based rehabilitation—a randomised controlled trial. J Clin Med. 2023;12:2074. 36902868 10.3390/jcm12052074 PMC10004240

[36] Scillia AJ , Issa K , Boylan MR , McDermott JD , McInerney VK , Patel DV , et al. Inpatient cruciate ligament reconstruction in the United States: a nationwide database study from 1998 to 2010. Orthopedics. 2016;39:e196–e202. 26726975 10.3928/01477447-20151222-18

[37] Smith TO , Davies L . A systematic review of bracing following reconstruction of the anterior cruciate ligament. Physiotherapy. 2008;94:1–10.

[38] Smith TO , Postle K , Penny F , McNamara I , Mann CJV . Is reconstruction the best management strategy for anterior cruciate ligament rupture? A systematic review and meta‐analysis comparing anterior cruciate ligament reconstruction versus non‐operative treatment. Knee. 2014;21:462–470. 24238648 10.1016/j.knee.2013.10.009

[39] Soma Y , Mutsuzaki H , Yoshioka T , Kubota S , Iwai K , Shimizu Y , et al. Muscle strength and efficiency of muscle activities recovery using single‐joint type hybrid assistive limb in knee rehabilitation after anterior cruciate ligament reconstruction. J Clin Med. 2023;12:6117. 37834760 10.3390/jcm12196117 PMC10573596

[40] Swirtun LR , Jansson A , Renström P . The effects of a functional knee brace during early treatment of patients with a nonoperated acute anterior cruciate ligament tear: a prospective randomized study. Clin J Sport Med. 2005;15:299–304. 16162987 10.1097/01.jsm.0000180018.14394.7e

[41] Vadalà A , Iorio R , De Carli A , Argento G , Di Sanzo V , Conteduca F , et al. The effect of accelerated, brace free, rehabilitation on bone tunnel enlargement after ACL reconstruction using hamstring tendons: a CT study. Knee Surg Sports Traumatol Arthrosc. 2007;15:365–371. 17149647 10.1007/s00167-006-0219-2

[42] Wright RW , Haas AK , Anderson J , Calabrese G , Cavanaugh J , Hewett TE , et al. Anterior cruciate ligament reconstruction rehabilitation: MOON guidelines. Sports Health. 2015;7:239–243. 26131301 10.1177/1941738113517855 PMC4482298

[43] Yang XG , Feng JT , He X , Wang F , Hu YC . The effect of knee bracing on the knee function and stability following anterior cruciate ligament reconstruction: a systematic review and meta‐analysis of randomized controlled trials. Orthop Traumatol Surg Res. 2019;105:1107–1114. 31279767 10.1016/j.otsr.2019.04.015

[44] Yapıcı F , Gür V , Fatih Sarı İ , Köksal A , Yurten H , Üçpunar H , et al. Prescription of knee braces after anterior cruciate ligament reconstruction: fact or fiction? Turk J Phys Med Rehab. 2022;68:355–363. 10.5606/tftrd.2022.8906 PMC9706800 36475105

[45] Zaffagnini S . Return to sport after ACL reconstruction: how, when and why? A narrative review of current evidence. Joints. 2015;3:25–30. 26151036 PMC4469040

